# High on Cannabis and Calcineurin Inhibitors: A Word of Warning in an Era of Legalized Marijuana

**DOI:** 10.1155/2016/4028492

**Published:** 2016-08-09

**Authors:** Naomi Hauser, Tanmay Sahai, Rocco Richards, Todd Roberts

**Affiliations:** Department of Medicine, Roger Williams Medical Center, Boston University School of Medicine, Providence, RI 02908, USA

## Abstract

Tacrolimus, a potent immunosuppressant medication, acts by inhibiting calcineurin, which eventually leads to inhibition of T-cell activation. The drug is commonly used to prevent graft rejection in solid organ transplant and graft-versus-host disease in hematopoietic stem cell transplant patients. Tacrolimus has a narrow therapeutic index with variable oral bioavailability and metabolism via cytochrome P-450 3A enzyme. Toxicity can occur from overdosing or from drug-drug interactions with the simultaneous administration of cytochrome P-450 3A inhibitors and possibly P-glycoprotein inhibitors. Tacrolimus toxicity can be severe and may include multiorgan damage. We present a case of suspected tacrolimus toxicity in a postallogeneic hematopoietic stem cell transplant patient who was concurrently using oral marijuana. This case represents an important and growing clinical scenario with the increasing legalization and use of marijuana throughout the United States.

## 1. Introduction

Tacrolimus is an immunosuppressive calcineurin-inhibiting medication commonly used in allogeneic hematopoietic stem cell transplant (HSCT) patients to prevent severe graft-versus-host disease (GVHD) [1–3]. There is a narrow therapeutic window and close clinical monitoring and laboratory monitoring are important to prevent toxicity. Supratherapeutic blood concentrations can result in an array of nonspecific adverse effects, which include hypertension, nephrotoxicity, severe tremor, hemolytic uremic syndrome, leukoencephalopathy, and coma [1, 2, 4, 5]. An increase in nephrotoxicity and neurotoxicity has been seen at blood concentrations greater than 20 ng/mL without any significant improvement in rate of GVHD [6–8]. Specific toxic side effects of tacrolimus, namely, tremor, may be idiosyncratic, however, rather than dose-dependent, as was concluded at a 1998 consensus conference convened to review tacrolimus use and effects.

It is important to recognize genetic variability and other exogenous factors that may alter the metabolism of tacrolimus and increase or decrease the level of tacrolimus in the blood. The P-glycoprotein efflux pump plays a large role in tacrolimus absorption from the gut and distribution in other tissues, while cytochrome P-450 3A (CYP3A) enzyme is primarily responsible for tacrolimus metabolism [9–11]. Different CYP3A alleles seem to be directly related to tacrolimus dose requirement and drug clearance [9]. Similarly, CYP3A inhibition by exogenous factors may increase the level of tacrolimus in the blood [12]. Well-known drugs with such effects include several macrolide antibacterials and triazole antifungals and preemptive tacrolimus dose-reduction has been proposed when drugs are to be administered concomitantly [3, 12]. Exogenous cannabinoids are another group of chemicals that similarly inhibit CYP3A [13]. Additionally, cannabinoids from marijuana have been shown to significantly inhibit the function of the P-glycoprotein transporter, which has a major role in tacrolimus absorption from the gut and distribution to other tissues [10, 11].

This P-glycoprotein and CYP3A inhibition by cannabinoids brings up the possibility for drug interactions and potential toxicity, particularly at a time of expanding medical marijuana laws throughout the country. We present a case of tacrolimus toxicity secondary to supratherapeutic drug levels in a postallogeneic HSCT patient using inhaled and edible marijuana.

## 2. Case Report

The patient is a 67-year-old man with relapsed follicular lymphoma, initially diagnosed and treated 10 years earlier, who was admitted on Day −7 for a matched-related allogeneic HSCT. He was conditioned with fludarabine, cyclophosphamide, and total body irradiation and started on acyclovir, levofloxacin, and posaconazole for antimicrobial prophylaxis. His pretransplant hospital course was uneventful and his transplant was uncomplicated. He was started on a continuous tacrolimus infusion drip at 1.8 mg/kg on transplant Day −2 with a goal serum tacrolimus level of 8–12 ng/mL, which is our bone marrow transplant unit's accepted therapeutic range. The patient's blood tacrolimus level was measured and the drip was decreased to 1.5 mg/kg and then to 1.0 mg/kg due to levels being persistently just above target. On Day +10 the patient admitted to taking edible marijuana gummies brought in by a family member and a urine toxicology screen was positive for tetrahydrocannabinol (THC). On Day +14 blood tacrolimus level on 1.0 mg/kg continuous infusion was therapeutic and the patient was transitioned to 1 mg twice daily oral tacrolimus. On Day +20 a second urine toxicology screen again returned positive for THC. Blood tacrolimus levels spiked to 43.8 ng/mL the following day and tacrolimus dose was cut in half to 0.5 mg twice a day. Despite the dose decrease, tacrolimus level continued to increase, peaking at 45.8 ng/mL on Day +23, and tacrolimus was held. As the patient's tacrolimus level climbed, he also started to show signs of likely tacrolimus toxicity. He developed diarrhea, body stiffness, tremors, and altered mental status, although there was no notable kidney function impairment. On Day +24 the patient was transferred to the intensive care unit (ICU) due to altered mental status and apparent increasing respiratory effort. Posaconazole was discontinued due to the potential for this antifungal to inhibit tacrolimus metabolism and tacrolimus was held. After three days in the ICU, the patient's mental status returned to near baseline and he was transferred back to the bone marrow transplant unit.

A third urine toxicology screen was done on Day +28 and returned negative for THC. Daily blood tacrolimus level had been checked and tacrolimus administration was held until Day +31, for a total of 10 days, when the level came down to within the accepted therapeutic range, at which time administration of both tacrolimus and posaconazole was resumed.

## 3. Discussion

As of December 2015, 23 states have passed medical marijuana laws and 3 states have legalized recreational use of the drug [14]. Additionally, marijuana usage among adults in the US more than doubled from 4.1% in 2001/2002 to 9.5% in 2012-2013 [15]. Because of this, it is important to keep in mind the likelihood of both inpatients and outpatients using marijuana and to understand the potential clinically significant drug interactions of exogenous cannabinoids, which are known to inhibit P-glycoprotein and CYP3A [10, 11, 13].

It should also be noted that the coadministration of the triazole antifungal ketoconazole with Δ9-tetrahydrocannabinol (THC)/cannabidiol (CBD) oromucosal spray (Sativex®, nabiximols) has been observed to increase cannabinoid concentration in the blood [16]. Our allogeneic HSCT patient was receiving the triazole posaconazole for antifungal prophylaxis while on tacrolimus. While this combination of tacrolimus with a triazole is common and is known to cause increased tacrolimus blood levels, the additional use of another CYP3A inhibitor likely compounded the drug interactions and complicated titration. In addition, it is suggested that cannabinoids may have immunosuppressive effects on their own via activation of cannabinoid receptor 2 [17]. This could imply that, along with altering serum tacrolimus levels, measured tacrolimus levels may not accurately reflect the degree of immune suppression when the two drugs are used simultaneously. It is also important to recognize the allelic variability of CYP3A in order to appreciate the unpredictable metabolism of tacrolimus, even in the absence of other exogenous drugs.

Research should be undertaken to elicit the exact nature and clinical impacts of the pharmacological interaction of exogenous cannabinoids in bone marrow transplant patients. The P-glycoprotein and CYP3A inhibition pose a serious concern for the need to regulate or at least monitor cannabis use in patients receiving tacrolimus after HSCT. Furthermore, there may be a benefit to obtaining a urine toxicology screen on these patients before starting the immunosuppressant tacrolimus in order to better adjust dosage and predict serum concentration.

## Abbreviations

HSCT: Hematopoietic stem cell transplant
GVHD: Graft-versus-host disease
CYP3A: Cytochrome P-450 3A
ICU: Intensive care unit
THC: Tetrahydrocannabinol.
