# Corrigendum to “High on Cannabis and Calcineurin Inhibitors: A Word of Warning in an Era of Legalized Marijuana”

**DOI:** 10.1155/2018/7095846

**Published:** 2018-09-06

**Authors:** Naomi Hauser, Tanmay Sahai, Rocco Richards, Todd Roberts

**Affiliations:** Department of Medicine, Roger Williams Medical Center, Boston University School of Medicine, Providence, RI 02908, USA

In the article titled “High on Cannabis and Calcineurin Inhibitors: A Word of Warning in an Era of Legalized Marijuana” [1], there was an error in the units used in the dose of continuous infusion rate of tacrolimus. Accordingly, in the “Case Report” section, “He was started on a continuous tacrolimus infusion drip at 1.8 mg/kg on transplant Day −2 with a goal serum tacrolimus level of 8–12 ng/mL, which is our bone marrow transplant unit's accepted therapeutic range. The patient's blood tacrolimus level was measured and the drip was decreased to 1.5 mg/kg and then to 1.0 mg/kg due to levels being persistently just above target” should be corrected to “He was started on a continuous tacrolimus infusion drip at 1.8 mg/24 hours on transplant Day −2 with a goal serum tacrolimus level of 8–12 ng/mL, which is our bone marrow transplant unit's accepted therapeutic range. The patient's blood tacrolimus level was measured and the drip was decreased to 1.5 mg/24 hours and then to 1.0 mg/24 hours due to levels being persistently just above target.”
